# Supplementary material for: Mapping hybrid functional-structural connectivity traits in the human connectome
Source: Netw Neurosci. 2018 Sep 1;2(3):306–22. doi: 10.1162/netn_a_00049 (PMC6145853; doi:10.1162/netn_a_00049)
Supplement: Supplementary file 1 [file netn-02-306-s001.pdf]

Amico, E. & Goñi, J. (2018). Supporting information for "Mapping hybrid functional-structural connectivity traits in the human connectome." *Network Neuroscience* 2(3), 306-322. https://doi.org/10.1162/netn_a_00049

Supplementary Materials

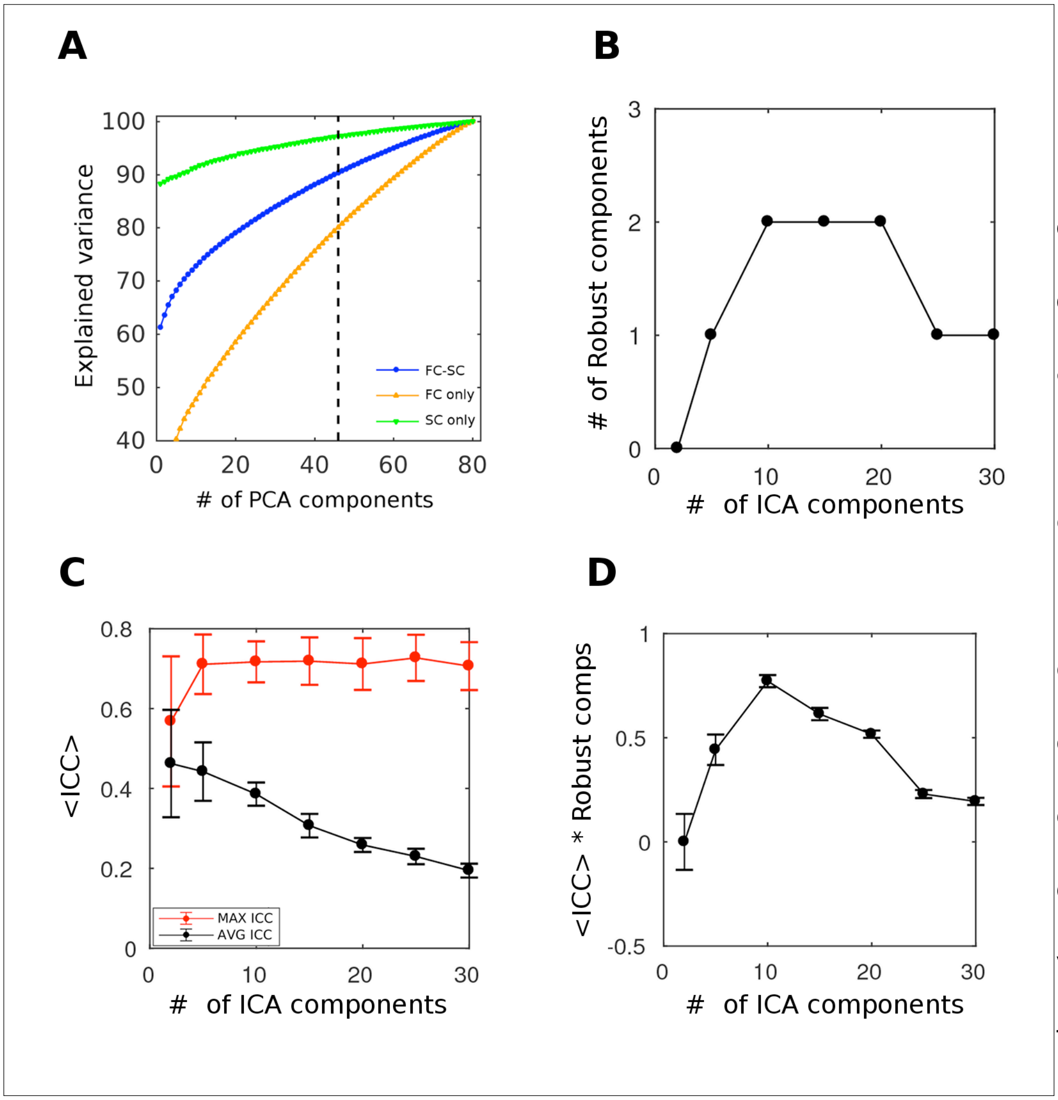

Fig. S1. Robustness evaluation of hybrid connICA. A) The cumulative explained variance for different components (from 1 to 80, being 80 the number of subjects (bootstrap, 100 runs, see Methods for details). Blue curve denote the cumulative explained variance for the joint FC-SC matrix, disentangled into FC (orange curve) and SC (green curve). The standard deviation across runs (not shown) was always lower than 1.5%. The dashed vertical black line represents the PCA cutoff at 90% of the variance on the joint scree plot (blue curve), at 45 components. Note that this cutoff on the joint FC-SC hybrid matrix captures about 95% of the

variance on SC and about 80% of the variance of FC. **B)** ICA exploration was performed on the PCA reconstructed dataset to check the range with the highest number of robust hybrid traits (see Methods). **C)** Also, ICA exploration was performed in order to check the IC range that would maximize task intra-class correlation on the weights of the hybrid traits (see Methods). **D)** The function chosen to pick the optimal number of independent component was defined as the product between number of robust components and avg ICC value in the ICA set. In this study we set the optimal number of IC is to 10 accordingly.

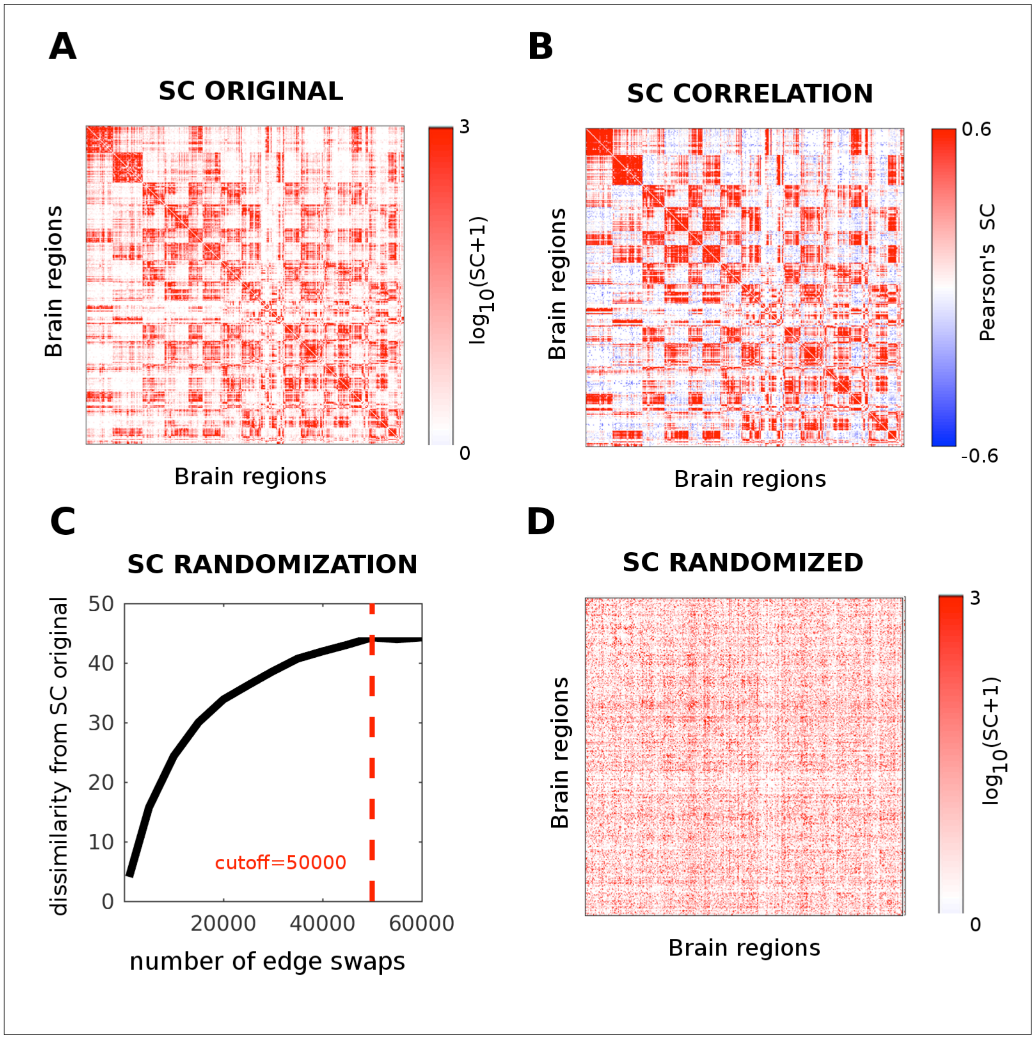

Fig. S2. Illustration of the different structural connectivity configurations employed. We started from one original individual structural connectome, SC (A); we then used for the hybrid

connICA the spatial correlation matrix obtained from the original SC (B); we tested results of the procedure against driving forces related to FC by randomizing the original SC through edge swapping (C); the number of changes was fixed to 25000 swaps, since it provides a trade-off between minimum number of swaps and maximum gain in dissimilarity with the original SC (see Methods). Finally, the spatial correlation of the randomized SC patterns (D) was computed and inputed in the hybrid connICA.

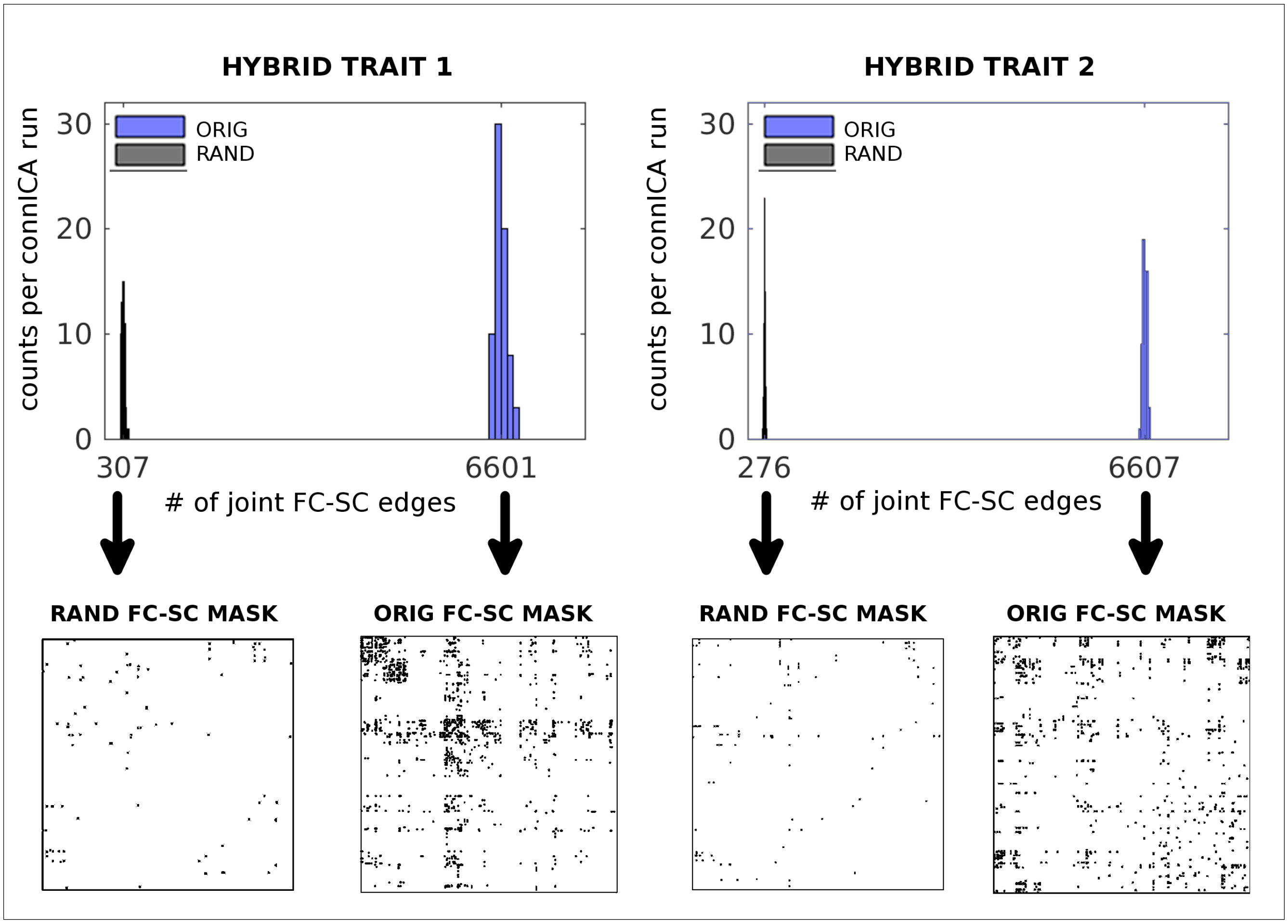

Fig. S3. Comparisons between original hybrid traits and SC-randomized ones. Top: For each of the 100 connICA runs, we counted the number of joint FC-SC edges in the mask (see also Fig.3), for the two original robust traits (blue histograms) and the two obtained after randomization of the structural connectomes (black histograms). Bottom: the joint masks obtained from the product of the more extreme values (outside the [5,95] percentile range) for the traits with randomized SC, and the original ones. Note how the number on nonzero elements significantly drops, hence making impossible the recovery of the hybrid circuitry depicted in Fig. 3 without the real structural connectivity profiles.

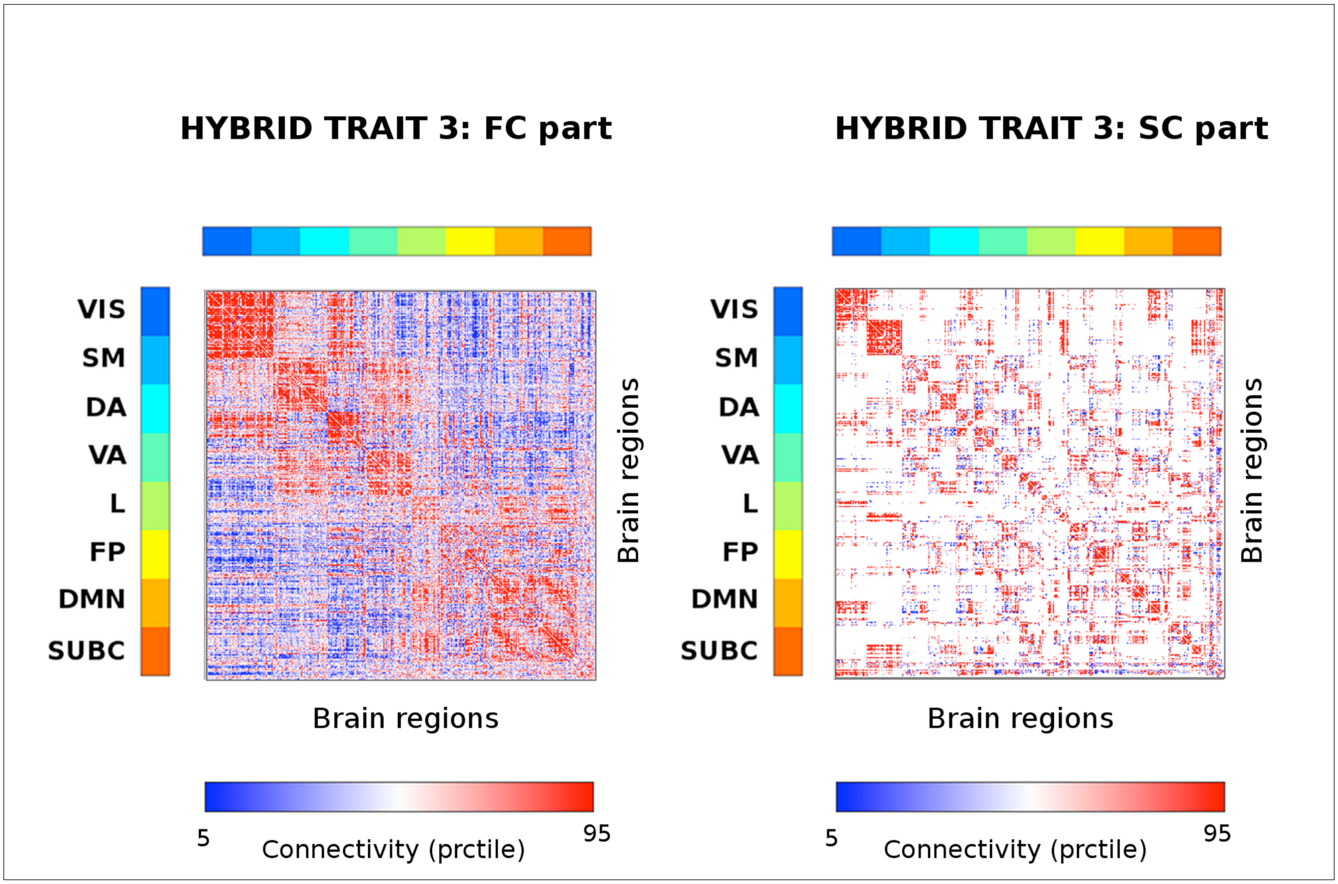

Fig. S4. The third (non task-dependent) robust hybrid trait found by hybrid connICA . The third robust trait extracted by hybrid connICA. Even if not related to task switching, it is representative of the intrinsic functional structural architecture of a human brain, since it mainly encompasses all the resting state networks functional blocks (left side of the plot), as well as the corresponding within network structural connections (right side of the plot).

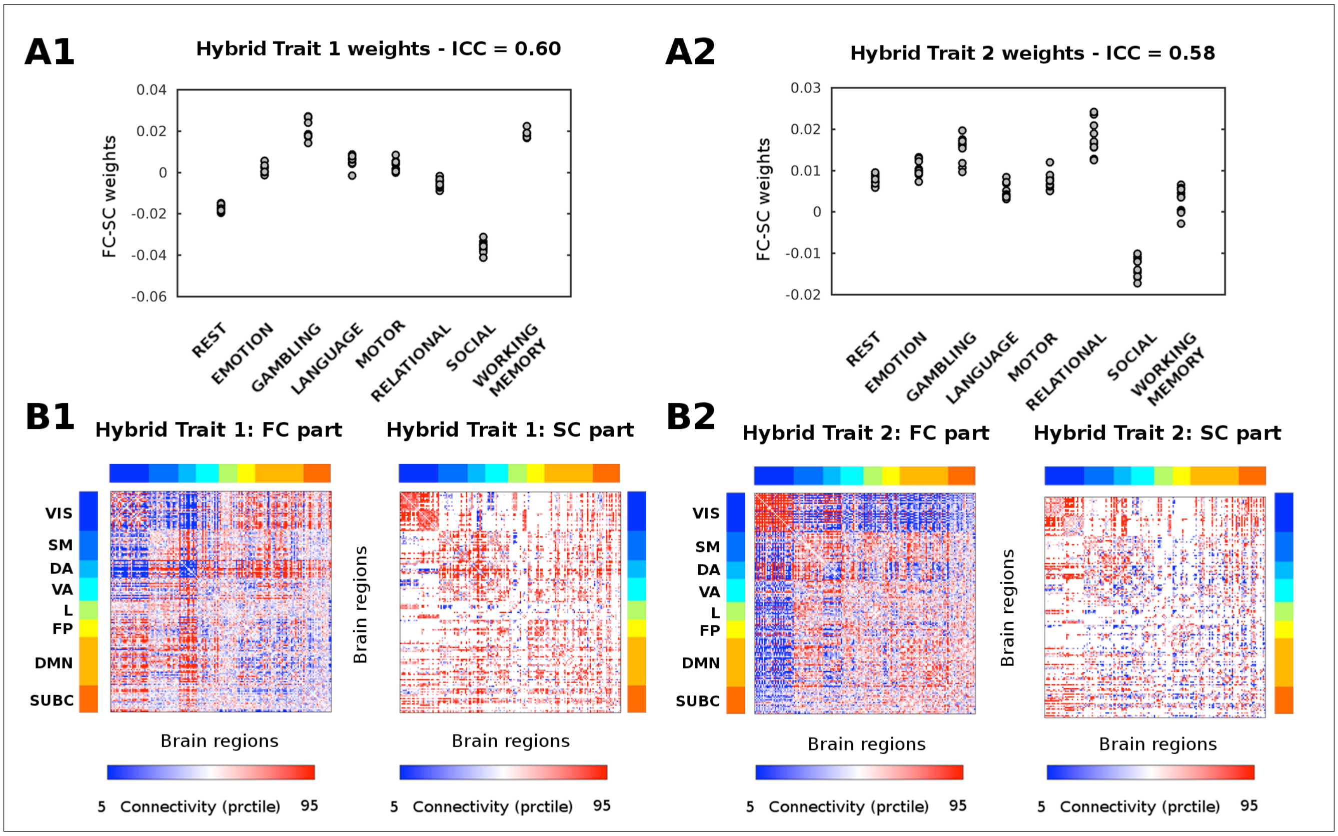

Fig. S5 Mapping of the main task-sensitive hybrid traits (Destrieux atlas, 164 brain regions). A1-A2) Quantified presence of each hybrid trait on each individual functional connectome. Subject weights are grouped according to each of the 7 tasks and resting state (10 subjects per task and resting state, see Methods). Task based intra-class correlation values are reported on top. **B1-B2) Visualization of the two averaged hybrid traits associated to significant changes (as measured by ICC) between tasks and resting state.** For ease of visualization, the hybrid traits are split in two matrices, corresponding to the functional connectivity (FC) and structural connectivity (SC) patterns. The brain regions are ordered according to functional RSNs: Visual (VIS), Somato-Motor (SM), Dorsal Attention (DA), Ventral Attention (VA), Limbic system (L), Fronto-Parietal (FP), Default Mode Network

(DMN), and for completeness, also subcortical regions (SUBC). Note that very similar traits were found when using a fine-grained parcellation (see Fig. 2, main manuscript).
